# Ribociclib and Palbociclib Are Safe and Effective for Treating Metastatic Breast Cancer: A Retrospective Study

**DOI:** 10.1002/cnr2.70630

**Published:** 2026-07-23

**Authors:** Cheng‐Wei Chou, Yi‐Hung Lo, Chia‐I. Wei, Chiann‐Yi Hsu, Chih‐Chiang Hung, Yen‐Dun Tzeng, Chia‐Hua Liu

**Affiliations:** ^1^ Division of Medical Oncology, Department of Oncology Taichung Veterans General Hospital Taichung Taiwan; ^2^ Department of Post‐Baccalaureate Medicine, College of Medicine National Chung Hsing University Taichung Taiwan; ^3^ Department of Surgery Kaohsiung Veterans General Hospital Kaohsiung Taiwan; ^4^ Department of Surgery Pingtung Veterans General Hospital Pingtung Taiwan; ^5^ Biostatistics Task Force, Department of Medical Research Taichung Veterans General Hospital Taichung Taiwan; ^6^ Division of Breast Surgery, Department of Surgery Taichung Veterans General Hospital Taichung Taiwan; ^7^ Department of Applied Cosmetology, College of Human Science and Social Innovation Hung Kuang University Taichung Taiwan; ^8^ School of Medicine, College of Medicine National Sun Yat‐sen University Kaohsiung Taiwan

**Keywords:** hormone receptor‐positive breast cancer, palbociclib, real‐world data, ribociclib

## Abstract

**Background:**

Cyclin‐dependent kinase (CDK) 4/6 inhibitors are standard for treating hormone receptor‐positive metastatic breast cancer when combined with endocrine therapy. However, comparisons of first‐line treatments are scarce.

**Aims:**

In this retrospective study, we aimed to determine the effects and safety of ribociclib and palbociclib as first‐line treatments for metastatic hormone receptor‐positive breast cancer in real‐world settings at two tertiary medical centers.

**Methods and Results:**

This retrospective observational study, conducted in two tertiary medical centers, included patients receiving palbociclib or ribociclib in combination with endocrine therapy. Treatment responses were assessed by imaging studies according to RECIST 1.1 criteria. The primary endpoint was 24‐month progression‐free survival (PFS). Safety outcomes were also recorded and compared between the treatment groups. The median follow‐up times for the ribociclib and palbociclib groups were 19.7 and 24.6 months, respectively. The 24‐month PFS rates were comparable between the ribociclib and palbociclib cohorts (55.4% vs. 55.2%, respectively; hazard ratio [HR] = 1.110, 95% confidence interval [CI]: 0.605–2.035; *p* = 0.736), but the objective response rate was significantly higher in the palbociclib group than in the ribociclib group (43.2% vs. 30.2%, *p* = 0.035). A high rate of dose reduction was observed in both cohorts due to treatment‐related toxicities (41.2% in the palbociclib group vs. 47.2% in the ribociclib group). The adverse effect profiles were comparable, although the incidence of grade 1 fatigue was slightly higher in the ribociclib group than in the palbociclib group (*p* = 0.043).

**Conclusion:**

In Taiwanese real‐world practice, ribociclib and palbociclib demonstrated comparable 24‐month efficacy. Our findings show that dose reductions do not compromise targeted treatment efficacy, supporting proactive toxicity‐guided dose to optimize patient tolerability without sacrificing oncological outcomes in Asian populations.

## 1 Introduction

Breast cancer (BC) is the most prevalent type of cancer diagnosed among Asian women, approximately 18% of all cancer cases, and the incidence has recently increased [1, 2]. Over two‐thirds of BCs are hormone receptor‐positive (HR+) [3]. Cyclin‐dependent kinases 4 and 6 (CDK4/6) are key mediators of cell growth, cell cycle, and crucial regulators in hormone‐positive BC [4]. Frontline treatment with CDK4/6 inhibitors combined with anti‐estrogen drugs and aromatase inhibitors (endocrine therapies) has significantly improved progression‐free survival (PFS) compared with endocrine therapy alone in HR+ and HER2‐negative metastatic BC [5, 6, 7, 8]. Thus far, only ribociclib combined with endocrine therapy as first‐line treatment has provided a significant overall survival benefit in prospective clinical trials such as MONALEESA‐2 and MONALEESA‐7 [9, 10]. However, the absence of head‐to‐head comparison trials and heterogeneity among studies has complicated evaluations of their outcomes.

Although BC shares similar risk factors globally, tumor biology and epidemiology differ between Asian and non‐Asian patients [11, 12, 13]. Premenopausal HR+ BC is more prevalent in Asian women than in Western women [14]. Given interethnic variations in genetic backgrounds, drug tolerability, and safety profiles, Asian and Western populations might distinctly differ [13]. Only 15%–30% of Asian patients have been included in trials of CDK4/6 inhibitors. A recent meta‐analysis has found a more pronounced PFS benefit for Asian patients than for non‐Asian patients (hazard ratio [HR] and 95% confidence interval [CI]: 0.39; 0.29–0.51, vs. 0.62; 0.54–0.71) [15]. However, the PFS benefit of palbociclib plus letrozole was similar between Asian and North American patients in the PALOMA‐4 trial [16].

The global application of CDK4/6 inhibitors such as palbociclib, ribociclib, and abemaciclib has significantly increased since they became approved. Recent real‐world evidence (RWE) underscores the advantages of combining CDK4/6 inhibitors with endocrine therapy in treating HR+ metastatic BC [17, 18]. While prospective trials like MONALEESA‐2 and MONALEESA‐7 have demonstrated a superior overall survival benefit for ribociclib [9, 10], existing cross‐trial comparisons are limited by trial heterogeneity, and direct head‐to‐head randomized trials are currently lacking. Furthermore, although several large‐scale comparative RWE studies have been published, they are overwhelmingly derived from Western registries (such as the US Flatiron Health database) [19]. These Western‐centric data cannot be seamlessly extrapolated to Asian populations due to critical interethnic variations. Asian patients frequently experience more pronounced hematological toxicities, especially neutropenia associated with CDK4/6 inhibitors, which often leads to a higher frequency of dose reductions and treatment interruptions in real‐world Asian practice [20].

However, with more real‐world data having been reported recently, showing similar clinical benefits. Real‐world data comparison in Asian were still limited. Whether these distinct tolerability profiles, dose titrations, and subsequent treatment compliance variations influence the comparative real‐world effectiveness of ribociclib versus palbociclib in Asian women remains a major knowledge gap. All three agents have received approval in Taiwan. Among these agents, palbociclib and ribociclib are fully reimbursed under the National Health Insurance (NHI) program with stringent, standardized criteria. This unique, centralized healthcare framework effectively eliminates the confounding effects of socioeconomic status and drug affordability on clinical outcomes, providing an exceptional and homogenous environment for comparative research. Against this backdrop, this retrospective study assessed the real‐world effectiveness and safety profiles of these CDK4/6 inhibitors by analyzing data from two tertiary medical centers, aiming to compare the effectiveness of both drugs in Asian patients.

## 2 Methods

### 2.1 Patient Selection

This study was a retrospective and observational cohort study. Data were electronically retrieved from the clinical databases of two tertiary medical centers in Taiwan: Taichung and Kaohsiung Veterans General Hospitals, regarding treatments administered between November 2018 and August 2022 and follow up until January 31, 2023.

Patients were considered eligible for inclusion in this retrospective cohort study if they met the following criteria: (1) histologically or cytologically confirmed HR+ and human epidermal growth factor receptor 2‐negative (HER2−) advanced, recurrent, or metastatic BC, where HER2 negativity was defined as an immunohistochemistry (IHC) score of 0 or 1+, or an IHC score of 2+ with a negative fluorescence in situ hybridization (FISH) result; (2) received either palbociclib or ribociclib in combination with endocrine therapy (including aromatase inhibitors or fulvestrant) as their first‐line (frontline) targeted regimen for metastatic disease within the designated study period; (3) were aged ≥ 20 years at the time of treatment initiation; and (4) at least > 30 days of follow up. Patients were excluded for any of the following reasons: no histological confirmation of breast adenocarcinoma, pathology reports indicating positive HER‐2 status or triple‐negative BC, lost to follow up, inadequate clinical records, human immunodeficiency virus (HIV) infection, not treated with a CDK4/6 inhibitor, and death within 30 days after diagnosis. The Ethics Committees of Taichung and Kaohsiung Veterans General Hospitals approved this study (approval IDs: CE21406B and KSVGH23‐CT5‐14, respectively). Both IRBs waived the requirement for informed consent owing to the retrospective study design. This multicenter retrospective cohort study was designed, conducted, and is reported in strict accordance with the Strengthening the Reporting of Observational Studies in Epidemiology (STROBE) guidelines for cohort studies [21].

### 2.2 Statistical Analysis

Patient characteristics are shown as medians with first and third quartiles (interquartile range; IQR) for continuous variables and numbers (*n*) with ratios (%) for categorical variables. The primary endpoint of this study was the 24‐month PFS rate, defined as the estimated proportion of patients who remained alive and free from objective disease progression at 24 months following the initiation of CDK4/6 inhibitor therapy. Time‐to‐event for PFS was calculated from the date of treatment initiation to the date of documented radiographic progression (per RECIST v1.1 criteria), clinical progression determined by the treating oncologist, or death from any cause, whichever occurred first. Patients who were alive and progression‐free at the 24‐month landmark or at their last follow‐up prior to 24 months were censored accordingly.

Secondary endpoints included the overall survival (OS, defined as the time from treatment initiation to death from any cause), objective response rate (ORR), disease control rate (DCR), and safety and tolerability profiles graded according to the Common Terminology Criteria for Adverse Events (CTCAE).

We estimated PFS and OS using Kaplan–Meier curves and differences were assessed using log‐rank tests. Categorical variables were compared using Fisher's exact tests and continuous variables were analyzed using independent *t*‐tests or chi‐square tests, depending on the nature of the data. Variables with clinical relevance or those showing a trending association in univariate analysis were entered into a multivariate Cox proportional hazards regression model to calculate adjusted hazard ratios (aHRs) and their 95% CIs to control for baseline confounding factors. All tests were two‐sided. Values with *p* < 0.05 were considered statistically significant.

## 3 Results

### 3.1 Characteristics of the Patients

**FIGURE 1:**
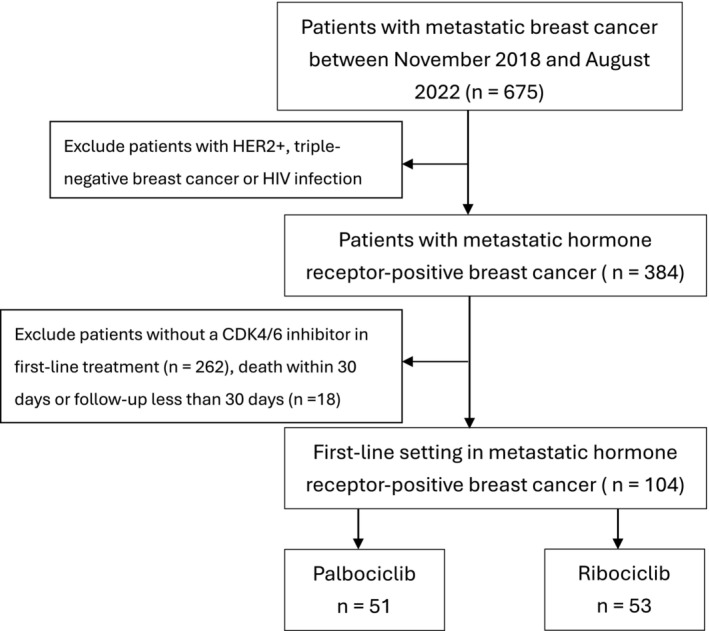
Flow diagram of selected patients with metastatic hormone receptor‐positive breast cancer treated with cyclin‐dependent kinase (CDK) 4/6 inhibitors.

During the study period, a total of 675 patients with metastatic BC from two tertiary medical centers were screened for eligibility. Among them, 384 patients were identified with HR+ disease. After sequentially excluding individuals who did not receive CDK4/6 inhibitor therapy, those who received these agents beyond the first‐line setting, or those with a treatment duration of less than 30 days following treatment initiation, we enrolled 104 patients with metastatic BC who received first‐line treatment with either ribociclib (*n* = 53) or palbociclib (*n* = 51) combined with endocrine therapy (Figure 1). The median follow‐up times for the ribociclib and palbociclib groups were 19.7 and 24.6 months, respectively.

The median age of the entire patient cohort at the time of treatment was 60.3 (IQR, 51–69) years (Table 1). Patients were significantly older in the palbociclib group than in the ribociclib group (median age: 63.7 vs. 56.9 years; *p* = 0.004). More patients were pre‐ or peri‐menopausal at the time of diagnosis in the ribociclib group than in the palbociclib group (20.8% vs. 15.7%; *p* = 0.011). The baseline characteristics of the two groups were largely comparable. Approximately 50% of patients were diagnosed with de novo metastasis and hormone therapies combined with CDK4/6 inhibitors comprised tamoxifen, aromatase inhibitors, and selective estrogen receptor degraders.

**TABLE 1:** Characteristics of patients with metastatic breast cancer treated with ribociclib and palbociclib.

	Total (*n* = 104)		Ribociclib (*n* = 53)		Palbociclib (*n* = 51)		*p*
Median age (years)	60.3	(51–69)	56.9	(47.5–67)	63.7	(55–71)	0.004^†^
ECOG							0.868
0	94	(90.4%)	48	(90.6%)	46	(90.2%)	
1	9	(8.7%)	4	(7.5%)	5	(9.8%)	
2	1	(1.0%)	1	(1.9%)	0	(0.0%)	
Disease status (metastasis)							0.216
De novo	54	(51.9%)	24	(45.3%)	30	(58.8%)	
Recurrent	48	(46.2%)	27	(50.9%)	21	(41.2%)	
Locoregionally recurrent	2	(1.9%)	2	(3.8%)	0	(0.0%)	
Metastatic sites							0.559
1	46	(44.2%)	24	(45.3%)	22	(43.1%)	
2	32	(30.8%)	18	(34.0%)	14	(27.5%)	
≥ 3	26	(25.0%)	11	(20.8%)	15	(29.4%)	
Menopausal status							0.011*
Postmenopausal	85	(81.7%)	42	(79.2%)	43	(84.3%)	
Premenopausal	12	(11.5%)	10	(18.9%)	2	(3.9%)	
Perimenopausal	7	(6.7%)	1	(1.9%)	6	(11.8%)	
Endocrine therapy							< 0.001^†^
Letrozole	68	(65.4%)	42	(79.2%)	26	(51.0%)	
Anastrozole	26	(25.0%)	6	(11.3%)	20	(39.2%)	
Fulvestrant	6	(5.8%)	5	(9.4%)	1	(2.0%)	
Exemestane	2	(1.9%)	0	(0.0%)	2	(3.9%)	
Tamoxifen	2	(1.9%)	0	(0.0%)	2	(3.9%)	

*Note:* Continuous and categorical data are expressed as medians with interquartile ranges (IQRs) and as numbers (*n*) with ratios (%), respectively.

Abbreviation: ECOG, Eastern Cooperative Oncology Group.

* *p* < 0.05 (chi‐square tests or Fisher exact tests).

^†^ *p* < 0.01 (chi‐square tests or Fisher exact tests).

### 3.2 Doses and Adjustments for CDK4/6 Inhibitors During Treatment of Metastatic HR+ BC

The initial ribociclib dose was predominantly the standard 600 mg in our cohort, except for one nonagenarian patient who began treatment with a conservative dose (200 mg). Conversely, the initial doses of palbociclib were the standard 125 mg for 39 (76.5%) and 100 mg for 12 (23.5%) patients (Table 2). The dose reduction rates were 47.2% and 41.2% in the ribociclib and palbociclib groups, respectively. The high dose reduction rates in our cohort were consistent with the notably higher dose reduction rate among the Asian population in the PALOMA‐2 study [22].

**TABLE 2:** Doses and effects of CDK4/6 inhibitors on metastatic hormone receptor‐positive breast cancer.

	Total (*n* = 104)	Ribociclib (*n* = 53)	Palbociclib (*n* = 51)		*p*
Initial dose (mg)					
Ribociclib					
600	52 (50.0%)	52 (98.1%)	0	(0.0%)	
200	1 (1.0%)	1 (1.9%)	0	(0.0%)	
Palbociclib					
125	39 (37.5%)	0 (0.0%)	39	(76.5%)	
100	12 (11.5%)	0 (0.0%)	12	(23.5%)	
Dose reduction					0.538
No	58 (55.8%)	28 (52.8%)	30	(58.8%)	
Yes	46 (44.2%)	25 (47.2%)	21	(41.2%)	
Best response					0.035*
CR	4 (3.8%)	3 (5.7%)	1	(2.0%)	
PR	34 (32.7%)	13 (24.5%)	21	(41.2%)	
SD	61 (58.7%)	32 (60.4%)	29	(56.9%)	
PD	5 (4.8%)	5 (9.4%)	0	(0.0%)	

Abbreviations: CR, complete response; PD, progressive disease; PR, partial response; SD, stable disease.

* *p* < 0.05 (Fisher exact tests).

### 3.3 Effects of CDK4/6 Inhibitors Against Metastatic HR+ BC

**FIGURE 2:**
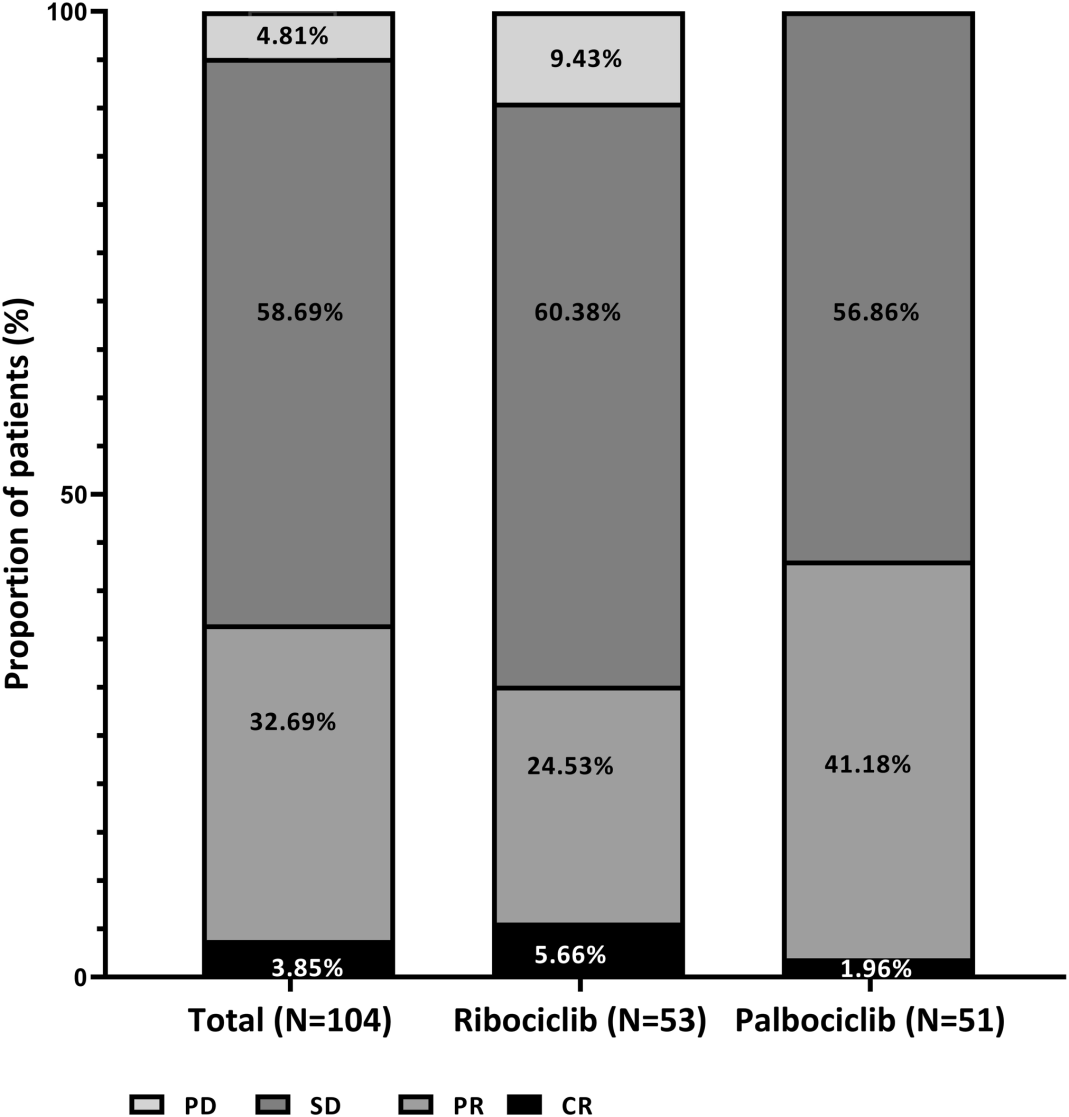
Best responses to cyclin‐dependent kinase (CDK) 4/6 inhibitors combined with hormone therapy to manage metastatic hormone receptor‐positive breast cancer.

**FIGURE 3:**
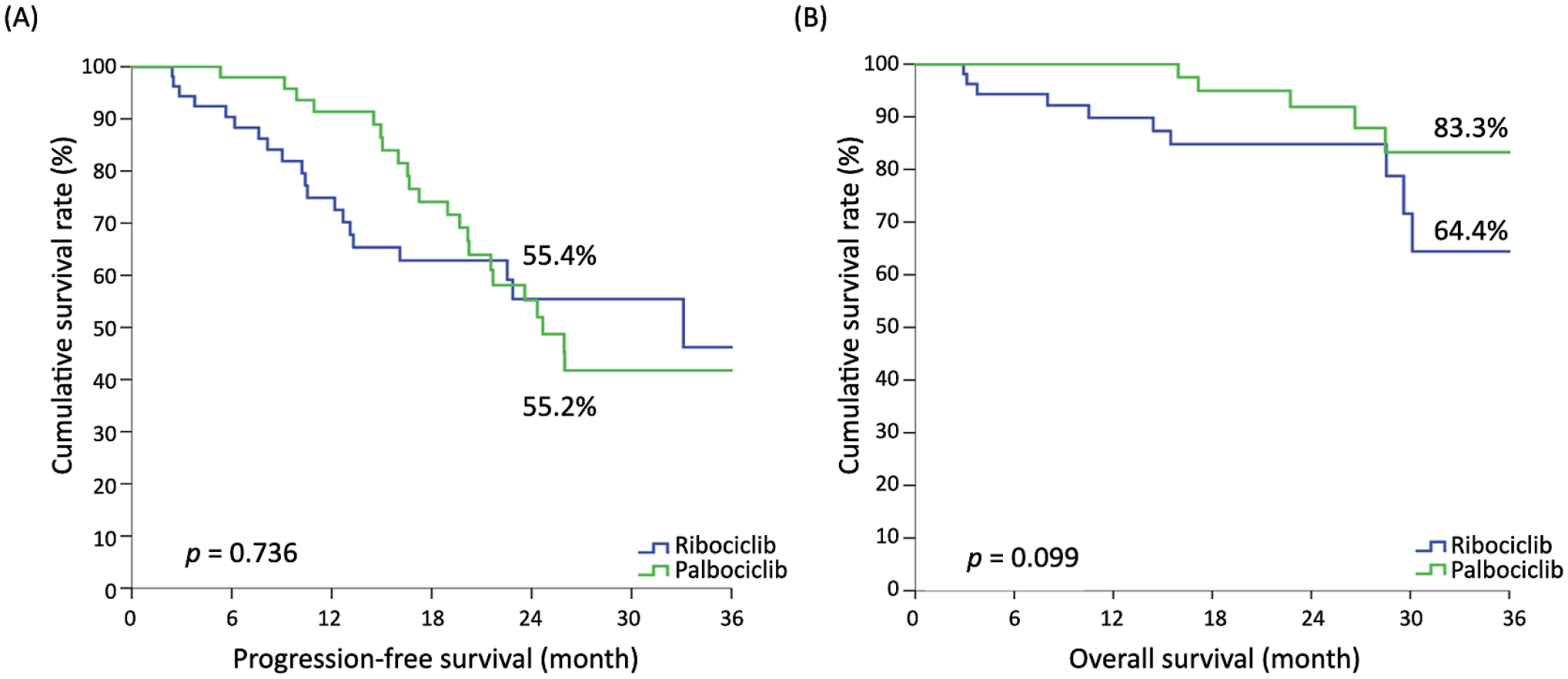
Effects of cyclin‐dependent kinase (CDK) 4/6 inhibitors in metastatic hormone receptor‐positive breast cancers are comparable. Kaplan–Meier survival curves for (A) progression‐free survival (PFS) and (B) overall survival (OS) of patients with metastatic breast cancer treated using CDK4/6 inhibitors. *p* < 0.05 (log‐rank tests).

With the reimbursement limitation of 24 months in Taiwan, we analyzed the best clinical response, 24‐month PFS, and OS rates to compare the effects of both CDK4/6 inhibitors in a frontline setting. ORRs to ribociclib and palbociclib were 30.2% versus 43.2% (*p* = 0.035), respectively (Table 2). The overall DCR was > 90% in both groups. Our cohort revealed significant clinical benefits in real‐world practice, similar to the result in PALOMA‐4 trial (Figure 2). Besides, the 24‐month PFS rates were comparable between the ribociclib and palbociclib cohorts (55.4% vs. 55.2%, respectively; HR = 1.110, 95% CI: 0.605–2.035; *p* = 0.736) (Figure 3A). The median OS was not reached in either group (Figure 3B).

### 3.4 Prognostic Factors for PFS

To identify potential prognostic factors for PFS and evaluate whether the baseline clinical imbalances confounded the therapeutic comparison, univariate and multivariate Cox proportional hazards regression analyses were performed (Table S1).

In the univariate analysis, the choice of CDK4/6 inhibitor was not a significant predictor of disease progression (univariate HR = 1.11, 95% CI: 0.60–2.03; *p* = 0.73). Factors significantly associated with an increased risk of progression in the univariate model included an ECOG performance status of 2 (HR = 12.78, 95% CI: 1.59–102.73; *p* = 0.01) and the locoregionally recurrent disease status (HR = 13.78, 95% CI: 2.82–67.32; *p* = 0.001). Variables with significant *p* values or clinical relevance were subsequently entered into the multivariate Cox regression model to control for potential confounding effects. The result demonstrated that only the locoregionally recurrent status remained an independent adverse prognostic factor for PFS, exhibiting a significantly higher risk of progression compared to de novo metastatic disease (multivariate HR = 14.34, 95% CI: 1.73–118.29; *p* = 0.01).

### 3.5 Adverse Effects of CDK4/6 Inhibitors

Regarding the adverse effects, both CDK4/6 inhibitors elicited a similar spectrum of adverse effects, with grade ≥ 3 neutropenia and leukopenia being the most frequent (Table 3). Neither CDK4/6 inhibitor caused treatment‐related mortality in our study. The incidence of fatigue, although higher in the ribociclib group than in the palbociclib group, was predominantly grade 1. No treatment‐related mortality emerged during our brief follow‐up period, which was consistent with the findings of controlled clinical trials [9, 23]. Therefore, the safety profiles of these inhibitors were validated.

**TABLE 3:** Adverse event profiles associated with CDK4/6 inhibitor treatment.

	Total (*n* = 104)		Ribociclib (*n* = 53)		Palbociclib (*n* = 51)		*p*
Neutropenia							0.413
None	41	(39.42%)	22	(41.51%)	19	(37.25%)	
Grade 1	6	(5.77%)	4	(7.55%)	2	(3.92%)	
Grade 2	10	(9.62%)	6	(11.32%)	4	(7.84%)	
Grade 3	40	(38.46%)	16	(30.19%)	24	(47.06%)	
Grade 4	7	(6.73%)	5	(9.43%)	2	(3.92%)	
Leukopenia							0.663
None	48	(46.15%)	26	(49.06%)	22	(43.14%)	
Grade 1	10	(9.62%)	4	(7.55%)	6	(11.76%)	
Grade 2	26	(25.00%)	13	(24.53%)	13	(25.49%)	
Grade 3	18	(17.31%)	8	(15.09%)	10	(19.61%)	
Grade 4	2	(1.92%)	2	(3.77%)	0	(0.00%)	
Anemia							0.131
None	86	(82.69%)	48	(90.57%)	38	(74.51%)	
Grade 1	7	(6.73%)	3	(5.66%)	4	(7.84%)	
Grade 2	7	(6.73%)	1	(1.89%)	6	(11.76%)	
Grade 3	3	(2.88%)	1	(1.89%)	2	(3.92%)	
Grade 4	1	(0.96%)	0	(0.00%)	1	(1.96%)	
Thrombocytopenia							0.204
None	98	(94.23%)	52	(98.11%)	46	(90.20%)	
Grade 1	2	(1.92%)	1	(1.89%)	1	(1.96%)	
Grade 2	2	(1.92%)	0	(0.00%)	2	(3.92%)	
Grade 3	2	(1.92%)	0	(0.00%)	2	(3.92%)	
Elevated liver enzymes							0.334
None	93	(89.42%)	50	(94.34%)	43	(84.31%)	
Grade 1	4	(3.85%)	1	(1.89%)	3	(5.88%)	
Grade 2	2	(1.92%)	0	(0.00%)	2	(3.92%)	
Grade 3	5	(4.81%)	2	(3.77%)	3	(5.88%)	
QT prolongation							1.000
None	103	(99.04%)	52	(98.11%)	51	(100.00%)	
Grade 1	1	(0.96%)	1	(1.89%)	0	(0.00%)	
Fatigue							0.043*
None	84	(80.77%)	40	(75.47%)	44	(86.27%)	
Grade 1	18	(17.31%)	13	(24.53%)	5	(9.80%)	
Grade 2	2	(1.92%)	0	(0.00%)	2	(3.92%)	
Headache							0.495
None	102	(98.08%)	51	(96.23%)	51	(100.00%)	
Grade 1	2	(1.92%)	2	(3.77%)	0	(0.00%)	
Diarrhea							0.678
None	98	(94.23%)	49	(92.45%)	49	(96.08%)	
Grade 1	6	(5.77%)	4	(7.55%)	2	(3.92%)	
Alopecia							0.495
None	101	(97.12%)	50	(94.34%)	51	(100%)	
Grade 1	1	(0.96%)	1	(1.89%)	0	(0%)	
Grade 2	2	(1.92%)	2	(3.77%)	0	(0%)	
Rash							0.468
None	92	(88.46%)	48	(90.57%)	44	(86.27%)	
Grade 1	7	(6.73%)	2	(3.77%)	5	(9.80%)	
Grade 2	4	(3.85%)	2	(3.77%)	2	(3.92%)	
Grade 3	1	(0.96%)	1	(1.89%)	0	(0.00%)	

* *p* < 0.05 (Fisher exact tests).

## 4 Discussion

Our study investigated the clinical effects and safety of combining ribociclib or palbociclib with endocrine therapy to manage metastatic HR+ BC. The effects did not substantially differ; the safety profiles were parallel, and PFS rates were similar between the groups. These findings emphasize the clinical value of CDK4/6 inhibitors for treating BC.

Current approved CDK4/6 inhibitors have significantly prolonged PFS compared with endocrine therapy alone, despite differences in pharmacology and kinase targets [24]. The PFS did not significantly differ in a matching‐adjusted indirect comparison of the MONALEESA‐2 and PALOMA‐2 trials, indicating similarly effective first‐line treatment as our findings [25]. In contrast, OS was not consistent in these trials [9, 23]. To date, only ribociclib as a first‐line treatment has conferred an OS benefit. However, real‐world studies have also supported an OS benefit of palbociclib [17, 26]. The ongoing HARMONIA SOLTI‐2101/AFT‐58 clinical trial is probably the first and sole prospective head‐to‐head comparison [27]. With the global approval of CDK4/6 inhibitors, more real‐world data have confirmed the similar clinical effectiveness of various CDK4/6 inhibitors.

Retrospective studies have compared the efficacy of different CDK4/6 inhibitors in a first‐line setting. For example, a nationwide real‐world comparative study in Qatar has revealed that the PFS and OS of palbociclib and ribociclib are similar, with the most prevalent side effect being neutropenia [28]. Real‐world clinical data did not reveal any significant differences among three available CDK4/6 inhibitors in Spain and the UK [29, 30]. The magnitude of the PFS benefit is significantly more dominant in the Asian population than in the non‐Asian populations [15]. Racial differences in tumor biology are an established issue for clinical trials [31, 32]. Real‐world data for Asian populations are scarce and predominantly limited to single‐agent studies [33, 34]. Herein, we compared the effects and toxicity of two CDK4/6 inhibitors in an Asian population in the real world. In Taiwan, the NHI reimbursement policy for CDK4/6 inhibitors is limited to 2 years with bone‐only disease excluded. This policy creates a distinctive artificial censoring environment at the 2‐year mark, which differs fundamentally from global randomized controlled trials where drug exposure is continuous until biological progression. This heterogeneous post‐reimbursement management introduces confounding factors into long‐term overall survival outcomes. Therefore, evaluating the 24‐month PFS rate as our primary endpoint provides the practical evaluation of real‐world comparative effectiveness between ribociclib and palbociclib in Taiwan. Despite this restriction, we were able to demonstrate that the 2‐year reimbursed use of both CDK4/6 inhibitors provides comparable clinical benefits for patients with metastatic HR+ BC during the reimbursement period. However, long‐term survival outcomes would be affected by subsequent treatment beyond the reimbursement policy.

The prevalence of de novo metastatic disease in pivotal trials is 34%–38% [5, 8]. In contrast, we found that 51.9% of patients presented with de novo metastatic disease, which exceeded the rates in clinical trials and was also slightly higher than real‐world data of 41% de novo diseases [17]. The ORR for palbociclib (43.2%) in the present study closely aligned with the 42.1% found in the PALOMA‐2 trial [5]. However, the ORR for ribociclib was 36.5% in the present study compared with 52.7% in the MONALEESA‐2 trial [8]. Nevertheless, the DCRs for both groups were more than 90%, providing clinically meaningful benefits.

Regarding dose reductions, a pivotal trial indicated rates of ~40% and ~58% in non‐Asian and Asian populations, respectively [15]. A pooled analysis found similar effectiveness when doses were modified owing to adverse events [35]. Real‐world data have suggested that dose reduction rates for non‐Asian populations vary between 30% and 50% [29, 36]. Another real‐world study from Taiwan also revealed a significant proportion of patients requiring dose reductions, yet it demonstrated clinical benefits of disease control comparable to those observed in clinical trials [37] We found that 44.2% of our patients required a dose reduction and 12.5% started at a lower dose. In other real‐world data from Asia, approximately 20% of patients start with doses below recommended levels [38]. Therefore, dose reductions were common but did not significantly compromise clinical effectiveness.

The adverse effects in our patients were similar to those found in previous clinical trials, with leukopenia being the most prevalent [6, 8]. Febrile neutropenia did not arise in either group during treatment with CDK4/6 inhibitors. Fatigue, although slightly more prevalent in the ribociclib group, was mostly grade 1. Therefore, the safety profiles of both CDK4/6 inhibitors were similar in real‐world practice.

A major and clinically reassuring finding of this multicenter study is that despite a high incidence of toxicity‐driven dose modifications, the real‐world efficacy of both palbociclib and ribociclib remained fully preserved. By confirming that the 24‐month PFS rate remains entirely uncompromised by dose reductions, our findings showed that we could successfully minimize toxicities and enhance patient compliance without sacrificing therapeutic efficacy.

Our study has several limitations. This retrospective analysis included data from only two medical centers, where prescribed endocrine therapy combinations varied among physicians. Menopausal status differed between the groups; the proportions of pre‐ or peri‐menopausal women were higher in the ribociclib group. The short follow‐up period coupled with the 2‐year reimbursement policy limit presents challenges to effective analyses of differences in OS. Besides, bone‐only disease patients were not included due to the reimbursement policy in Taiwan, which also affects the patients' characteristics.

We confirmed that first‐line treatment with either ribociclib or palbociclib combined with endocrine therapy maintained similar clinical efficacy and safety profiles, which were consistent with findings of previous clinical trials. Despite dose reductions in the rates of both drugs, the CDK4/6 inhibitors continued to deliver significant clinical benefits. This evidence supports their use as a first‐line treatment, reinforcing their valuable role in managing HR+ BC.

## Author Contributions

**Yi‐Hung Lo:** data curation. **Cheng‐Wei Chou:** conceptualization, writing – original draft, writing – review and editing, funding acquisition, visualization. **Chia‐I. Wei:** data curation. **Yen‐Dun Tzeng:** conceptualization, writing – review and editing, writing – original draft. **Chih‐Chiang Hung:** conceptualization, project administration, methodology, writing – review and editing. **Chiann‐Yi Hsu:** formal analysis, visualization. **Chia‐Hua Liu:** conceptualization, writing – original draft, writing – review and editing, data curation.

## Funding

This work was supported by Taichung Veterans General Hospital, TCVGH‐1123701C.

## Ethics Statement

The Institutional Review Boards at Taichung Veterans General Hospital and Kaohsiung Veterans General Hospital approved this study (IRB nos. CE21406B and KSVGH23‐CT5‐14, respectively). Both IRBs waived the requirement for informed consent owing to the retrospective study design.

## Conflicts of Interest

The authors declare no conflicts of interest.

## Supporting information


**Table S1:** Summary of univariate and multivariate analyses of progression‐free survival.

## Data Availability

The data that support the findings of this study are available on request from the corresponding author. The data are not publicly available due to privacy or ethical restrictions.
