# Supplementary material for: Ribociclib and Palbociclib Are Safe and Effective for Treating Metastatic Breast Cancer: A Retrospective Study
Source: Cancer Rep (Hoboken). 2026 Jul 23;9(7):e70630. doi: 10.1002/cnr2.70630 (PMC13393296; doi:10.1002/cnr2.70630)
Supplement: Supplementary file 1 — Table S1: Summary of univariate and multivariate analyses of progression‐free survival. [file CNR2-9-e70630-s001.docx]

| **Supplementary Table 1. Summary of univariate and multivariate analyses of progression-free survival** | | | | | |
| --- | --- | --- | --- | --- | --- |
| Patient Characteristics | Univariate | | Multivariate | | |
|  | HR (95% CI) | *P* value | HR (95% CI) | *P* value |  |
| **Age** |  |  |  |  |  |
|  | 0.99(0.96-1.01) | 0.44 |  |  |  |
| **ECOG** |  |  |  |  |  |
| 0 | Reference |  |  |  |  |
| 1 | 1.92 (0.80-4.57) | 0.14 |  |  |  |
| 2 | 12.78(1.59-102.73) | 0.01^*^ | 1.14(0.07-18.44) | 0.92 |  |
| **Disease status (metastasis)** |  |  |  |  |  |
| De novo metastatic | Reference |  |  |  |  |
| Metastatic recurrent | 1.26 (0.68-2.35) | 0.45 |  |  |  |
| Locoregionally recurrent | 13.78(2.82-67.32) | 0.001^*^ | 14.34(1.73-118.29) | 0.01^*^ |  |
| **Metastatic sites** |  |  |  |  |  |
| 1 | Reference |  |  |  |  |
| 2 | 0.98(0.46-2.06) | 0.96 |  |  |  |
| > 3 | 1.61(0.79-3.27) | 0.18 |  |  |  |
| **Menopausal status** |  |  |  |  |  |
| Postmenopausal | Reference |  |  |  |  |
| Premenopausal | 0.81(0.31-2.07) | 0.66 |  |  |  |
| Perimenopausal | 0.71(0.21-2.35) | 0.58 |  |  |  |
| **CDK4/6 inhibitor** |  |  |  |  |  |
| Palbociclib | Reference |  |  |  |  |
| Ribociclib | 1.11(0.60-2.03) | 0.73 |  |  |  |
| ECOG, Eastern Cooperative Oncology Group. *p<0.05. | | | | | |
